# Vagally Associated Second Degree Atrio-Ventricular Block in a Dog with Severe Azotemia and Evidence of Sympathetic Overdrive

**DOI:** 10.3390/vetsci9050223

**Published:** 2022-05-05

**Authors:** Radu Andrei Baisan, Andreea Cătălina Turcu, Eusebiu Ionuț Condurachi, Vasile Vulpe

**Affiliations:** Clinics Department, Faculty of Veterinary Medicine, Iasi University of Life Sciences “Ion Ionescu de la Brad”, Mihail Sadoveanu Alley no. 8, 700489 Iași, Romania; baisan.andrei_mv@uaiasi.ro (R.A.B.); sebi.condurachi@gmail.com (E.I.C.); vvulpe@uaiasi.ro (V.V.)

**Keywords:** arrhythmia, autonomic nervous system, canine, electrocardiography, heart rate variability

## Abstract

A 14 years old, 6 kg, mix-breed male dog with severe azotemia due to urinary bladder herniation was presented to our Veterinary Teaching Hospital (VTH). Electrocardiography revealed normal heart rate of 100 bpm, evidence of sinus respiratory arrhythmia (SRA) and frequent second degree atrio-ventricular block following peak inspiratory phase suggestive of vagally-induced atrio-ventricular conduction delay. Echocardiographic examination showed mild mitral regurgitation without any other cardiac changes, and systolic (SAP) and diastolic (DAP) blood pressure values were 185/90 mmHg (SAP/DAP). Cardiac troponin I (cTnI) was increased to 7.3 ng/mL, suggesting a myocardial injury. A Holter examination revealed evidence of overall decrease in heart rate variability with evidence of sympathetic overdrive on time and frequency domain as well as when the non-linear Poincaré plot was analyzed. Based on the author’s knowledge, this is the first report of a second degree atrio-ventricular block associated with vagal activity in a dog, with evidence of sympathetic overdrive and severe azotemia.

## 1. Introduction

Respiratory-associated atrio-ventricular (AV) block is a very rare finding and only a few cases have been described in human medicine [1,2,3]. Second degree AV block is characterized by an intermittent interruption of atrio-ventricular conduction and can be classified as physiologic, during situations of high vagal tone, or pathologic, resulting from degenerative, inflammatory or neoplastic processes [4]. Vagally mediated AV block is defined as a paroxysmal first, second or third degree block preceded or associated with slowing of the sinus rate [5] and is most likely to be reflex induced in origin, mediated by vagal effects on both the sinus and AV node [6]. In this paper, we present a case of second-degree AV block associated with the respiratory phase in a dog with azotemia due to urinary bladder herniation and evidence of myocardial injury. One of the first reports in human medicine of respiratory-associated second-degree AV block hypothesized that the vagal activity varying with respiration, inhibiting the atrio-ventricular conduction, could be the responsible mechanism for this conduction abnormality [1]. However, to the best of the author’s knowledge, this association was not reported in veterinary medicine. Therefore, the aim of this report is to describe the clinical findings of a dog with azotemia secondary to urinary bladder herniation, evidence of sympathetic overdrive and respiratory-associated second-degree AV block and to speculate on the underlying mechanism.

## 2. Case Description

A 14 years old, 6 kg, mix-breed male dog was presented to our VTH for apathy and loss of appetite for several days. The dog was previously diagnosed with a perianal mass suspected to be an intestinal loop or urinary bladder herniation. At the time of presentation, the dog was unable to stand and was unresponsive. Physical examination revealed normal body condition score, pink mucosal membranes and capillary refilling time < 2 s. Inspection of the thorax revealed normal respiratory pattern, auscultation revealed normal respiratory sounds and the respiratory rate was 13 breaths per minute. Cardiac auscultation revealed a II/VI low intensity holosystolic murmur with left apical point of maximal intensity, with a rate of 100 beats per minute. No therapy was administered prior to presentation in our clinic.

Based on the clinical findings, the dog was subjected to blood analysis, ultrasonography and complete cardiologic examination. Written consent for all procedures was obtained from the owner. Blood analyses revealed increased blood urea nitrogen (BUN) (>180 mg/dL, normal ranges 7–25 mg/dL), increased creatinine (12.7 mg/dL, normal ranges 0.3–1.4 mg/dL), low albumin (2.4 g/dL, normal ranges 2.5–4.4 g/dL), low total calcium (7.9 mg/dL, normal ranges 8.6–11.8 mg/dL) and increased potassium (6.1 mmol/L, normal ranges 3.6–5.8 mmol/L). Alkaline phosphatase, alanine aminotransferase, blood glucose and total protein were within normal ranges. Complete blood count revealed mildly increased number of neutrophils (12.23 × 10^3^/μL, normal ranges 3–12 × 10^3^/μL), decreased number of RBC (3.92 × 10^6^/μL, normal ranges 5.5–8.5 × 10^6^/μL), decreased hemoglobin (8.1 g/dL, normal ranges 12–18 g/dL) and low hematocrit (28%, normal ranges 37–55%). Analysis of cardiac biomarkers showed markedly increased cTnI (7.3 ng/mL, normal < 0.03 ng/mL) and normal brain natriuretic peptide (NTproBNP) (530 pmol/L, normal < 900 pmol/L).

The blood pressure was measured with an oscillometric device (VET HDO, S + B medVet, Babenhausen, Germany) using a C1 size cuff from the tail and the mean of five measurements was 185/90 mmHg (SAP/DAP).

Ultrasound focused examination revealed an anechoic thin-walled structure with a small quantity of free fluid around it, localized in the perianal region confirming the herniation of the urinary bladder. This structure was punctured under ultrasonographic guidance and 300 mL of dark yellow fluid consistent with urine was removed.

**Figure 1:**
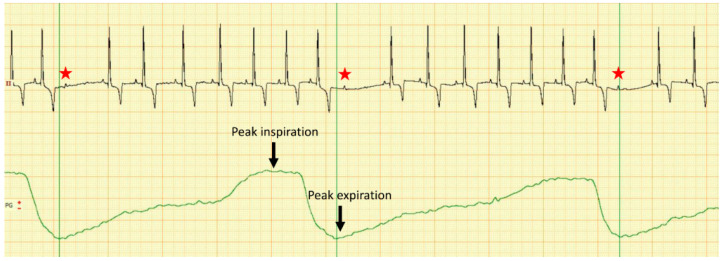
Dual channel recording of the electrocardiogram in lead II (upper line) and respiratory curve (lower green line) in a dog with severe azotemia. Note that there is a repetitive second-degree 2:1 AV block (red star) following each inspiration peak with a variable delay; calibration 10 mm/mV, 50 mm/s.

Furthermore, the dog was subjected to a cardiologic examination consisting of a 5 min 6-lead electrocardiography with overlapped respiratory curve recorded through an air-flow sensor placed 1 cm in front of the nostrils (PolySpectrum 8E/X, Ivanovo, Russia) and a trans-thoracic echocardiography (Logiq V5 General Electric Medical System, Wuxi, China) from both right and left parasternal views, as previously described [7]. No therapy was administered at the time of cardiologic examination. Electrocardiography revealed a predominant sinus rhythm with evidence of sinus respiratory arrhythmia showing the gradual increase and decrease of heart rate associated with the respiratory cycle, with a heart rate of 100 bpm, presence of wandering pacemaker and a mean electrical axis of 83°. The electrocardiographic measurements were within normal ranges. During the 5-min recording, there were 46 AV blocks following the peak-inspiration with a delay mean time from the end of the inspiratory peak to the onset of the blocked P-wave of 681 milliseconds (minimum time-142 ms; maximum time-1020 ms), as presented in Figure 1.

**Figure 2:**
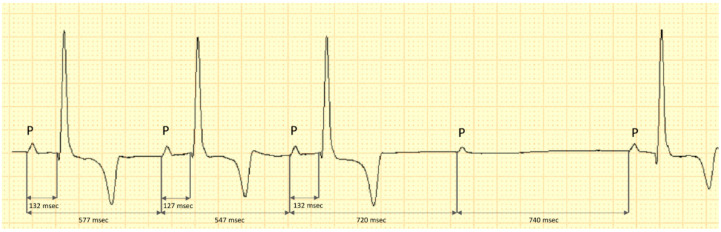
Capture of the electrocardiogram in lead II of a dog with severe azotemia. The fourth P-wave is blocked and no associated QRS complex is visible. There is no evidence of PR interval prolongation before the blocked P-wave, however, a P–P prolongation is present prior and after the occurrence of the bock. This may be indicative of the vagal activity on the sinus node, decreasing the atrial rate after inspiration phase; calibration 10 mm/mV, 50 mm/s.

The respiratory second-degree Mobitz type II non-advanced 2:1 associated AV block was constant, repetitive and did not show a PQ interval prolongation prior to the blocked P-wave. However, there was a P–P interval prolongation before and after the block occurrence (Figure 2).

Echocardiography showed a normal aspect of all the atrio-ventricular and arterial leaflets, with a mild regurgitating jet over the mitral valve with a maximum velocity of 2.8 m/s. The left atrium to aorta ratio was 1.26 (normal < 1.6) [8], and the LA antero-posterior diameter was 1.97 (normal 1.87–2.81) [9]. The dimension of the left ventricle in systole and diastole indexed to body-weight were 0.62 (normal 0.71–1.26) and 1.43 (normal ranges 1.27–1.85), respectively. The interventricular septum and posterior wall in diastole indexed to BW were 0.42 (normal ranges 0.29–0.59) and 0.46 (normal ranges 0.29–0.6) [10], respectively, and the shortening fraction of the LV calculated by the Teicholz formula was 54.7%. Spectral Doppler interrogation of the aortic and pulmonary flows revealed a laminar aspect with normal maximum velocities (0.97 m/s for the pulmonary artery flow and 1.1 m/s for the aortic flow).

Considering the blood creatinine concentration and the hypertensive status, the dog was classified as having chronic kidney disease (CKD) Stage 4, according to the IRIS staging [11]. The patient was admitted to the intensive care unit for stabilization and monitoring prior to the surgical repair of the hernia. The assigned therapy consisted of: 30 mL Ringer Lactate solution IV; Pantoprazole (1 mg/kg IV); Amoxicillin (15 mg/kg IM); Buprenorphine (0.02 mg/kg IV). The owner consented to a Holter monitoring during this time. The dog did not respond to therapy therefore a worsening of general status and level of consciousness were observed.

A Holter monitoring system was installed and recorded for 4 h and 30 min until the dog developed two generalized epileptiform seizures, suspected to be induced by azotemia. These were attempted to be managed by administering Diazepam (1 mg/kg IV). Considering the severe prognosis, the owner requested euthanasia and no necropsy examination was available.

The analysis of the Holter monitoring revealed a mean heart rate of 90 bpm with a maximum of 113 bpm and 85 second-degree AV blocks more frequent at the start and the end of the recording. No other arrhythmias were detected on the Holter monitoring. The heart rate variability (HRV) analyses from the Holter recoding were performed with a free analysis software (Kubios v. 3.5.0, Kupio, Finland) [12] and revealed decreased values of time domain and frequency domain as shown in Table 1.

**Figure 3:**
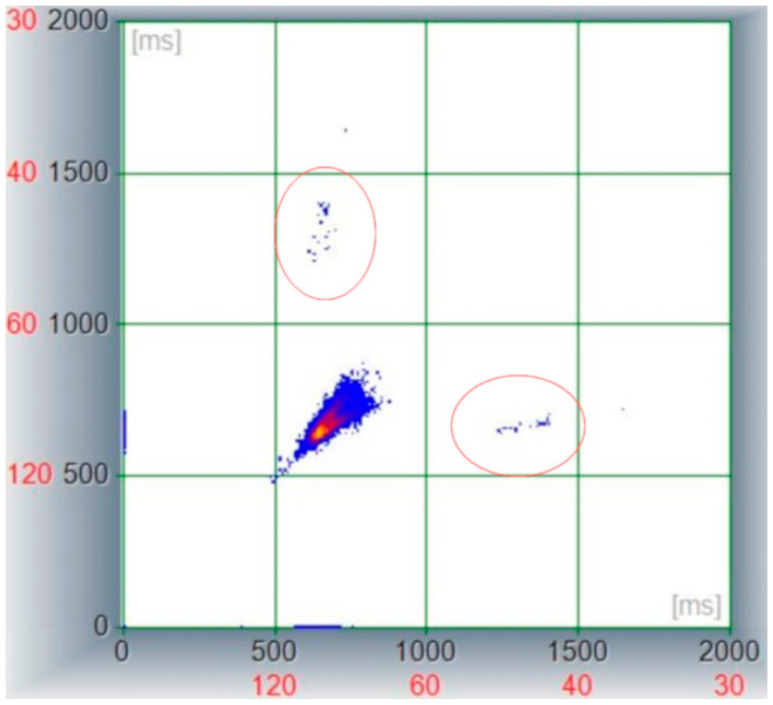
Poincaré plot derived from a 4.5 h Holter recording in a dog with severe azotemia. Note the central cluster lacking avoidance zone and showing a limited distribution among *X* and *Y* axes being stacked between 500 and 800 ms (representing a HR between 75 and 120 bpm); the two lateral clusters (encircled areas) are represented by RR pairs including the atrio-ventricular block where the software calculated the previous and following R waves as a “long RR” interval.

Similarly, the examination of the non-linear representation of beat-to-beat patterning expressed through the Poincaré plot revealed a reduced distribution of the consecutive RR interval coordinates with a torpedo shape [14], without avoidance zone [15] and two lateral separated clusters represented by the coordinates where the AV block occurred, as shown in Figure 3.

Based on the results of the examinations, the clinical diagnosis was urinary bladder herniation, severe azotemia and suspicion of myocarditis based on the troponin I blood concentration. In addition, based on the electrocardiographic, Holter recording and heart rate variability analysis, a vagally induced second-degree AV block and sympathetic overdrive were associated with the primary clinical diagnosis.

## 3. Discussion

This case report presents a dog with severe azotemia with evidence of sympathetic overdrive, myocardial injury and respiratory-associated second-degree AV block.

It is known that the sinus node is controlled by both sympathetic and parasympathetic input. During vagal activity the electrocardiographic manifestations include sinus rate slowing, sinus arrest and various degrees of AV blocks. One of the characteristic ECG findings of vagally mediated bradycardia is the simultaneous occurrence of AV block associated with sinus slowing [6]. Furthermore, vagal activation may have disparate effects on the sinus and AV nodes and may be accompanied by sympathetic overdrive [6]. However, the dog in the present report was also diagnosed with azotemia, which could represent the main reason for sympathetic overdrive [16].

In humans, kidney disease is associated with a general autonomic nervous system (ANS) imbalance which may also play a role in sudden cardiac death [17]. Vita et al. demonstrated in 30 uremic patients that 53% of the patients had autonomic dysfunction, in which 40% was isolated to the parasympathetic limb and 13% had combined parasympathetic and sympathetic damage [18]. Studies in veterinary medicine have also demonstrated that HRV is reduced in dogs with kidney failure compared to normal dogs, revealing a sympathetic overdrive [19,20].

Interestingly, despite the evidence of sympathetic overdrive in this dog, the heart rate was not increased and respiratory sinus arrhythmia was visible on the electrocardiographic tracing. Moreover, the evidence of vagal activity is supported by the prolongation of the P–P interval after the inspiration phase, even during the AV block occurrence. The sympathetic nervous system increases HR, by decreasing the time to produce an action potential in the sinus node. The parasympathetic nervous system is its antagonist and decreases HR, by increasing the time taken to produce an action potential in the phase 4 of sinus node. Vagal stimulus causes bradycardia, rapid and short duration modulation, controls rapid responses and increases heart rate variability [21]. This type of sinus automatism is related to respiratory phases and is called sinus respiratory arrhythmia, which is a periodic cardiorespiratory phenomenon characterized by heart rate acceleration during inspiration and heart rate deceleration during exhalation [22] and is usually associated with normal vagal tone activity [23].

Besides the presence of SRA in this dog, a repetitive second-degree AV block was present consistently after peak-inspiration. Vagal activation is known to simultaneously affect both the sinus and atrio-ventricular nodes. The resulting electrocardiographic (ECG) manifestations can include sinus rate slowing, sinus arrest and varying degrees of AV node blocks [6].

One study reported two cases of unusual reflex AV block in humans, which were not preceded by sinus rate decrease, however, in one case the arrhythmia was induced by stomach and duodenal insufflation during endoscopy, while in the second case it occurred during an episode of nausea, diaphoresis, retching followed by vomiting and extreme lightheadedness [6]. The first similar case was recorded in 1947 by Öhnell and Andersson in a man, describing a blocked beat occurring each respiratory cycle, however the PQ interval was increasing prior to the AV block [1]. The authors suggested that the underlying mechanism may be related to the vagal activity varying with respiration inhibiting the atrio-ventricular conduction. In another case report, a 46 years old woman presented with short episodes of AV blocks during inspiration which disappeared with the administration of atropine [3]. Based on the response to atropine, this arrhythmia was suspected to be caused by a vagal discharge during inspiration. Although, in the present case, this arrhythmia did not occur after a sinus rate decrease and we did not perform an atropine test because we suspected a sympathetic overdrive linked to the urologic condition, the association with the respiratory phase indicates a parasympathetic involvement.

This dog had also increased concentration levels of cardiac troponin I, suggesting myocardial injury. Experimentally-induced uremic cardiomyopathy in rats has led to reduced ischemic tolerance and larger myocardial infarctions [24]. These findings are a consequence of morphological changes in uremic myocardial tissue where the number of myocardial fiber decreases while their diameter increases, leading to increased oxygen demand of the uremic cardiomyocyte and a disturbed metabolic compensation during hypoxia [25]. In these conditions, the AV block may be explained by inflammation, direct mechanical compression or fibrosis, or both, resulting in a variable response to steroids [26]. Therefore, these changes of the myocardium may trigger conductions delays, such as AV blocks. Based on these findings, it is possible that in the present case, the AV node might have been more prone to autonomic changes due to the myocardial injury exhibiting vagally-induced conduction abnormalities. However, the effect of uremic cardiomyopathy on the AV node activity is not yet fully understood. Interestingly, there was evidence of sympathetic overdrive in the present case which, based on the current knowledge, should suppress the parasympathetic activity [27]. In a healthy heart, there is a dynamic relationship between the parasympathetic nervous system (PNS) and the sympathetic nervous system (SNS). Since these divisions can produce contradictory actions, such as speeding and slowing the heart, their effect on an organ depends on their current balance of activity. While the SNS can suppress PNS activity, it can also increase PNS reactivity. Increased PNS activity may be associated with a decrease, increase or no change in SNS activity [27]. However, the magnitude of interaction between the two branches of the ANS is not yet fully understood.

## 4. Conclusions

To the best of the author’s knowledge, this is the first report of respiratory-associated second-degree atrio-ventricular block in a dog with azotemia and sympathetic overdrive.

Severe azotemia secondary to urinary tract disorders may induce autonomic nervous system imbalance. Furthermore, uremic cardiomyopathy may sensitize the nodal tissue in the heart predisposing it to arrhythmias such as conduction delays. Therefore, these mechanisms may worsen the clinical state of the patient and should be carefully monitored.

## Figures and Tables

**Table 1:** Time and frequency domain heart rate variability values derived from 4.5 h Holter monitoring in a dog with kidney failure and reference values.

HRV Measurements	Resulted Values		Reference Ranges [13]
Time domain	SDNN (ms)	54	208.86 ± 77.1
	rMSSD (ms)	46	259 ± 120.17
	pNN50%	5.06	71.84 ± 13.96
Frequency domain	LF band (ms^2^)	212	1501.24 ± 736.32
	HF band (ms^2^)	704	5845.45 ± 2914.20
	LF/HF	0.3	0.28 ± 0.11

SDNN, standard deviation of all NN intervals; rMSSD, the root-mean-square of successive R-R interval differences; pNN50, the difference between consecutive R-R intervals which included the percentage of successive R-R intervals > 50 ms; LF, low frequency band expressed as units of spectral power; HF, high frequency band expressed as units of spectral power; LF/HF, low frequency to high frequency ratio.

## Data Availability

The data presented in this study are available in the manuscript.
